# The assessment of reliability and validity of the Thai Versions of the Thirst Distress Scale for patients with Heart Failure and the Simplified Nutritional Appetite Questionnaire in heart failure patients

**DOI:** 10.1177/17449871241292563

**Published:** 2024-12-14

**Authors:** Jenjiratchaya Thanapholsart, Ehsan Khan, Satit Janwanishstaporn, Porntipa Thongma, Saowanee Naowapanich, Pornpoj Pramyothin, Srisakul Chirakarnjanakorn, Porntera Sethalao, Thitipong Tankumpuan, Nana Waldréus, Geraldine A. Lee

**Affiliations:** PhD Candidate, Department of Adult Nursing, Florence Nightingale Faculty of Nursing, Midwifery and Palliative Care, King’s College London, London, UK; Senior Lecturer, Department of Adult Nursing, Florence Nightingale Faculty of Nursing, Midwifery and Palliative Care, King’s College London, London, UK; Assistant Professor, Cardiologist, Department of Cardiology, Faculty of Medicine, Siriraj Hospital, Mahidol University, Bangkok, Thailand; Assistant Director of Nursing for Risk Management in Nursing, Department of Nursing, Faculty of Medicine, Siriraj Hospital, Mahidol University, Bangkok, Thailand; Head of Quality Development Nursing Division, Department of Nursing, Faculty of Medicine, Siriraj Hospital, Mahidol University, Bangkok, Thailand; Assistant Professor, Nutritionist, Department of Nutrition, Faculty of Medicine, Siriraj Hospital, Mahidol University, Bangkok, Thailand; Assistant Professor, Cardiologist, Department of Cardiology, Faculty of Medicine, Siriraj Hospital, Mahidol University, Bangkok, Thailand; Medical Instructor, Cardiologist, Department of Cardiology, Faculty of Medicine, Siriraj Hospital, Mahidol University, Bangkok, Thailand; Assistant Professor, Faculty of Nursing, Mahidol University, Bangkok, Thailand; Assistant Professor, Division of Nursing, Department of Neurobiology, Care Sciences and Society, Karolinska Institutet, Stockholm, Sweden; Professor of Nursing and Chair of Health Service Research, Catherine McAuley School of Nursing & Midwifery, Brookfield Health Sciences Complex, University College Cork, Cork, Ireland

**Keywords:** appetite, heart failure, reproducibility of results, surveys and questionnaires, thirst

## Abstract

**Background:**

Heart failure (HF) patients can experience poor appetite and intense thirst. The Thirst Distress Scale for HF (TDS-HF) and the Simplified Nutritional Appetite Questionnaire (SNAQ) are tools used to assess these symptoms. However, these questionnaires are not currently available in Thai.

**Aims:**

To translate the TDS-HF and SNAQ into the Thai language and to evaluate the reliability and validity of these translated instruments.

**Methods:**

The TDS-HF and the SNAQ were translated from English to Thai using a forward–backward method. Their face and content validity, their internal consistency and test–retest reliability, were assessed with a sample of 20 HF patients recruited from a single supra-tertiary hospital in Thailand.

**Results:**

Participants reported that the TDS-HF and the SNAQ had face validity. The content validity was assessed as adequate by the expert panel. The Cronbach’s alphas of the TDS-HF and the SNAQ were 0.91 and 0.71, respectively. The intraclass correlation coefficients of the TDS-HF and the SNAQ were 0.91 and 0.91, respectively.

**Conclusions:**

The Thai versions of the TDS-HF and the SNAQ demonstrate adequate validity and reliability. These tools hold significant potential for supporting clinical practice, particularly for nurses involved in HF management by helping to assess thirst and appetite effectively.

## Introduction

In 2020, there were approximately 26 million people with heart failure (HF) worldwide (Lala et al., 2020); however, the specific prevalence of HF in Thailand is unknown (Ariyachaipanich et al., 2019). HF is generally caused by coronary artery disease and hypertension (McDonagh et al., 2021) leading to an elevation of neurohormonal activation which further aggravates HF, leading to a vicious cycle of further neurohormonal activation. Neurohormonal activation which is linked to an increase in Angiotensin II also causes low intestinal perfusion which manifests as gastrointestinal symptoms, such as poor appetite, nausea or vomiting and indigestion (Thanapholsart et al., 2023). Activation of these neurohormones can also lead to fluid overload and thirst (Holst et al., 2003) which can all impact on a patients’ quality of life (Allida et al., 2018; De Vecchis et al., 2016) and cause increased stress and anxiety (Waldréus et al., 2018a).

In addition to neurohormonal activation, people with HF who live in tropical countries could struggle with issues of thirst and appetite. Thailand for example sees high temperatures and humidity (e.g. a mean 27.5C and maximum of 42.4C; The Thai Meterological Department, 2021). These high temperatures could contribute to bodily fluid loss, whilst Thai food also has a high-salt content which can result in a subsequent increase in thirst. In addition, research suggests that if thirst associated with meal consumption cannot be satisfied, this can further decrease the amount of food consumed (Leib et al., 2016). It is therefore possible that in Thailand, people may experience excessive thirst which may also contribute to a lack of appetite.

There has been a greater focus in Thailand on using nutritional assessments (Sukkriang and Somrak, 2021), such as the Mini Nutritional Assessment (MNA) and Short Form-MNA (Nestlé Nutrition Institute, 2009); however, these do not specifically assess appetite. There are several widely used instruments, such as MNA and Visual Analogue Scale, available to assess thirst and appetite, including the Thirst Distress Scale for Patients with Heart Failure (TDS-HF) and the Simplified Nutritional Appetite Questionnaire (SNAQ). In particular, the TDS-HF and the SNAQ are suitable for use with people with HF as they are condition specific and have a short completion time (Waldréus et al., 2018b). Additionally, this study aims to translate these two instruments for use in further research to screen for cardiac cachexia – a condition characterised by muscle wasting and body weight loss associated with chronic HF – where the SNAQ is recommended to assess anorexia (Evans et al., 2008). However, these two questionnaires are not available in the Thai language.

Therefore, to allow more precise assessments of thirst and appetite in Thai people with HF, this study aimed to (1) translate and culturally adapt the TDS-HF and SNAQ into the Thai language and (2) examine the validity and reliability of these translated versions in a population of Thai people with HF.

## Method

### Study design

A qualitative approach through semi-structured interviews was used to examine the content and face validity of the translated questionnaires, and a quantitative approach was implemented to examine the reliability of the tools. The translation process of the questionnaires from English to Thai included forward translation, backward translation, finalising and reliability (pre) testing (Tsang et al., 2017). This process was performed following published guidance (Waldréus et al., 2018b) and professional advice from the author of the TDS-HF scale (Waldréus, personal communication).

### Instruments

The instruments used were (1) the English versions of the TDS-HF and the SNAQ, and (2) the Thai versions of the TDS-HF and the SNAQ.

The SNAQ is a tool recommended by the Cachexia Consensus for assessing anorexia in cachexia (Evans et al., 2008).It was developed from the longer Appetite, Hunger and Sensory Perception Questionnaire (AHSP) (Wilson et al., 2015), containing four questions that evaluate appetite and meal frequency, each rated on a 5-point scale (Lau et al., 2020; Wilson et al., 2005). The reliability of the SNAQ is supported by a reported Cronbach’s alpha of 0.77 (Andreae et al., 2015). The SNAQ scores range from 4 to 20, with scores below 14 indicating poor appetite.

The TDS-HF is developed to assess thirst distress in patients with HF on a 5-point Likert scale (Waldréus et al., 2018b). It includes eight items that address thirst-related problems like dry mouth and discomfort due to thirst. Scores range from 8 to 40 with higher scores indicating greater thirst distress. The original English version of the TDS-HF has a reported Cronbach’s alpha of 0.90 (Waldréus et al., 2018b).

### Translation process

#### Stage 1: Forward translation

There were three native Thai speakers who also spoke English involved in the forward translation process of the TDS-HF and SNAQ from English to Thai. These were (1) the lead researcher (JT) who has an expertise on thirst and HF, (2) a Thai academic instructor (TT) who is an expert in heart failure and (3) a linguist (PC).

JT and TT worked independently to translate the scales and then agreed to combine their translated versions of the questionnaires (Supplementary File). The forward translation of TDS-HF and SNAQ translated by the language expert (PC) differed from the combined version from JT and TT. Thus, two sets of Thai versions of the TDS-HF were sent for backward translation.

#### Stage 2: Backward translation

Two native English speakers (DW and MM) who are also fluent in Thai translated the TDS-HF and SNAQ (both versions) back from Thai to English. They did not refer to the original English version when translating and worked independently. The backward translation were sent to expert panel to determine which translated items more accurately reflected the original version (English version).

#### Stage 3: Finalisation of items

**Figure 1.**
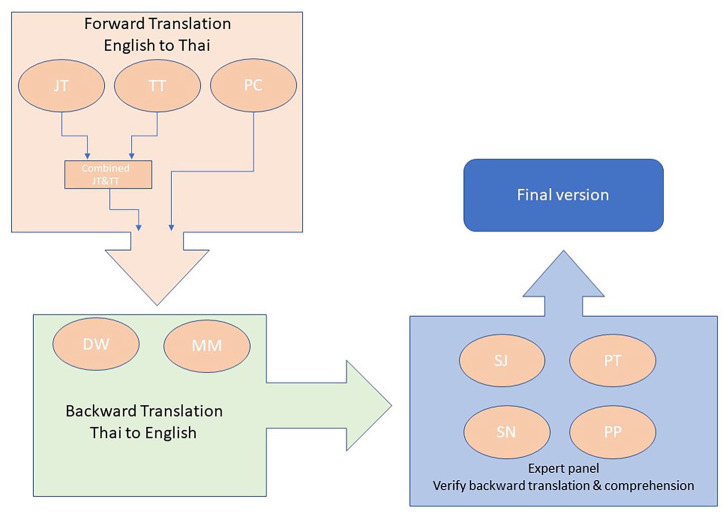
The diagram illustrates the translation process for the TDS-HF and the SNAQ. SNAQ, Simplified Nutritional Appetite Questionnaire; TDS-HF: Thirst Distress Scale for HF.

Any differences between the forward and backward translation of the TDS-HF and SNAQ were resolved by JT and the expert panel which comprised one cardiologist (SJ), one HF nurse (PT), one highly experienced cardiac nurse (SN) and one dietician (PP; Figure 1).

The expert panel also suggested a number of minor word changes to the translations which can be seen in Table 1.

**Table 1.** Minor word changes suggested by expert panel.

Change	Reason
TDS-HF	
A change of the word ‘distress’ to ‘affect’ in the instructions?	Experts felt that ‘Distress’ is seen as a medical term relating to a physical condition, such as ‘respiratory distress’, rather than a psychological condition which was the original meaning in the English version
The third item TDS-HF was changed from ‘sandpaper’ to ‘rough’ with ‘sandpaper’ in parenthesis.	The panel believed that Thai people would not refer to a dry mouth as like ‘sandpaper’ but say their mouth felt ‘rough’.
The eighth item was changed from ‘I feel that my thirst is too hard to overcome’ to ‘My thirst is hard to get over’	Experts felt that the use of the word ‘overcome’ could be subject to mixed interpretation in Thai.
SNAQ	
The word ‘of’ was added into the third item	To improve general readability
Replacement of a semi-formal word to eat with formal word to eat.	Thai writing features formal versions of the common words, and so a semi-formal word was replaced with a formal word.

SNAQ: Simplified Nutritional Appetite Questionnaire; TDS-HF: Thirst Distress Scale for patients with heart failure.

In addition, members of the project team suggested adding a descriptor for each of the Likert scale score points for increased clarity when choosing ratings. Once all the changes were made, the research team agreed the final versions of the questionnaires (Supplementary File).

#### Stage 4: Reliability and validity testing

The translated versions of the Thai TDS-HF and the SNAQ then underwent reliability and validity testing. The content validity of the scales, which looks at the relevance of items to the concept being measured (Tsang et al., 2017), was assessed by an expert panel containing four members as mentioned in stage 3. Face validity, which ensures that items appear valid to people who are likely to be assessed by an instrument (Tsang et al., 2017), was conducted using a sample of Thai people with HF through semi-structured interviews. This same sample provided data which allowed the test–retest reliability of the scales to measure their temporal stability. Internal consistency reliability testing, which ascertains how well each item measures the content of the assessment tool, was also calculated from this collected patient data. Further details regarding details method to collect data can be seen in data collection process.

### Setting and sample

A convenience sampling approach was employed to recruit participants in a HF clinic. Chronic patients from an outpatient HF clinic in a single-centre, supra-tertiary hospital in Bangkok, Thailand, were invited to take part. Given the recruitment setting, the baseline characteristics of eligible participants had diagnosed with chronic HF with an ejection fraction below 40% and/or advanced HF requiring comprehensive care, leading to their admission to the HF clinic.

The sample size needed for the test–retest reliability analysis was calculated based on the intraclass correlation coefficient (ICC) between test and retest, aiming to achieve a minimum reliability of 0.60 with power at 80% and alpha at 0.05 (Bujang, 2017; Bujang et al., 2024). Based on recommendations from two studies by Bujang et al., 14–15 participants would be sufficient to reach the desired ICC. However, the decision was made to over-recruit participants to account for potentially low response rates to the returned questionnaires. Since this study piloted the translated questionnaires, a large sample size was not deemed necessary. Hence, 20 participants were consented.

### Ethical consideration

Prior to conducting the study, ethical clearance was obtained from King’s College London (HR/DP-21/22-26225) and Siriraj Institutional Review Board, Faculty of Medicine, Mahidol University (certificate of approval no. Si 073/2022).

### Data collection process

#### Recruitment process

Each week, the HF nurse reviewed patients’ appointments and medical records to assess eligibility. The nurse then contacted eligible patients by phone before their appointment to inform them about the study. Those who were interested were sent the consent form and information sheet. Inclusion criteria for the study was (1) being 18 years or over and (2) attending the clinic with a diagnosis of chronic HF. Exclusion criteria included (1) an inability to provide informed consent or (2) being physiologically unstable with fluctuating symptoms of breathlessness, tachypnoea or tachycardia. Written consent was obtained prior to starting the data collection process of completing questionnaires and conducting interviews. Data were collected from April to June 2022.

#### Data collection process

Participants completed the TDS-HF and the SNAQ with a paper and pencil while they waited to see their consultant in the HF clinic. The HF nurse ensured that there were no unanswered items when participants returned completed questionnaires. Upon returning their questionnaires, participants received another copy from the nurse in a stamped addressed envelope to complete and return 1 week later. They were also interviewed virtually using the “LINE” app, a messenger app, (Line Corporation, 2022) by the researcher (JT) regarding their comprehension and cultural relevance of the language at the clinic. The duration of interviews was approximately 10–30 minutes. All participants were asked the following questions: (1) Do you have any thoughts on the questionnaires? (2) How have you scored each item? (3) Were there any questions that were not clear? (4) Were there any issues about the wording of the questions? JT reviewed all items in the TDS-HF and the SNAQ with participants, focusing on questions 2 and 4 to evaluate their understanding of the questionnaires and their rating of the items.

### Data analysis

As with the original development of the TDS-HF, content validity and face validity were examined (Waldréus et al., 2018b). Content validity was examined by an expert panel as defined in the translation process above. Face validity of the questionnaires was assessed by interviewing the participants. Given the straightforward nature of the interview questions aimed at exploring participants' understanding of the questionnaires, the primary interview responses were grouped and reported. Additionally, since a qualitative approach does not require a large sample size (Gerrish et al., 2015), 20 participants were deemed adequate.

The IBM SPSS statistical package Version 28 (IBM Corp, 2021) was used for analysing the quantitative data for reliability testing. Means, standard deviations, median and interquartile ranges (IQR) for the scales were calculated. Cronbach’s α was calculated to examine the reliability (internal consistency) of the Thai versions of the TDS-HF and the SNAQ. An acceptable Cronbach’s α was considered to be >0.60 (Raharjanti et al., 2022). The corrected item-total correlation was calculated to evaluate internal consistency of the questionnaires, and the acceptable value for each item was set at >0.30 (Raharjanti et al., 2022).

The test–retest reliability was assessed using ICC (Koo and Li, 2016). ICC and 95% confidence intervals were computed based on a mean of *k* measurements (*k* = 2), absolute agreement and two-way mixed effects. This meant that there were two measurements in this regard, as the test–retest reliability with replicated measurements cannot be considered as random samples (Koo and Li, 2016). Only the data from participants who completed the questionnaire twice were included for ICC calculation. The acceptable ICC value was >0.75 to indicate good reliability (Koo and Li, 2016).

### Additional data analysis

An intention-to-treat analysis was performed to account for missing data by using the pooled means using the multiple imputation method (MIM). The assumption of using MIM was that the data were missing at random, as the dataset included individuals with similar characteristics (Sterne et al., 2009), such as stable HF and normal cognitive function. Therefore, the observed data could be used to explain the missing data (Li et al., 2015). To calculate MIM, it is recommended to have less than 10% missing data, as exceeding this threshold could introduce bias into the multiple imputation process (Lee and Huber, 2021). The Cronbach’s α after a specific item had been deleted was also calculated when there was feedback that two or more questions substantially overlapped.

## Results

Twenty-five patients who were eligible were contacted by the HF nurse. Of these number, 20 patients expressed their interests and agreed to participate in this study. The average time taken to complete the TDS-HF and the SNAQ were 3.93 ± 2.41 minutes and IQR 1.97 minutes, respectively, and the median time was 3.66 minutes and 2.35 minutes, respectively. There was no missing data for the retest of the TDS-HF. However, there was missing data from two participants in the second administration of the SNAQ, accounting for 4.7% of the total responses from the 16 participants. Since this fell under the 10% threshold, MIM was used. The missing data included one response missing for item 2 and another participant missing responses for both items 2 and 4. These gaps were filled using MIM, with replacement values of 3.95 and 3.80, respectively.

The means of test vs retest of the TDS-HF and the SNAQ were 20.85 ± 7.17 vs 21.56 ± 5.75 and 14.65 ± 2.30 vs 15.29 ± 1.46, respectively. The Cronbach’s α of the TDS-HF and the SNAQ at the initial test and retest were 0.91 and 0.84 and 0.71 and 0.46, respectively (Table 2). These findings indicate good reliability with the exception of the Cronbach’s α for SNAQ (re-test). Both the TDS-HF and the SNAQ demonstrate excellent ICC.

**Table 2.** Frequency, Cronbach’s α and intraclass correlation coefficient of the TDS-HF and the SNAQ.

Questionnaires	Items (Thai and English versions)	Test (*n* = 20)					Retest (*n* = 16)					ICC (*n* = 16)
		Frequency (%)					Frequency (%)					
		1	2	3	4	5	1	2	3	4	5	
TDS-HF	1. อาการกระหายน้ำของฉันก่อกวนใจอย่างมาก (My thirst bothers me a lot)	2 (10)	8 (40)	6 (30)	3 (15)	1 (5)	3 (18.8)	3 (18.8)	6 (37.5)	3 (18.8)	1 (6.3)	First and second response Single measure 0.83 [95%CI 0.58–0.94] Average measure 0.91 [95% CI 0.74–0.97]
	2. ฉันรู้สึกไม่สุขสบายอย่างมากเมื่อฉันกระหายน้ำ (I am very uncomfortable when I am thirsty)	4 (20)	7 (35)	4 (20)	3 (15)	2 (10)	1 (6.3)	4 (25)	8 (50)	2 (12.5)	1 (6.3)	
	3. ปากของฉันรู้สึกแห้งสาก (เหมือนกระดาษทราย)เมื่อฉันมีอาการกระหายน้ำ (My mouth feels like sandpaper when I am thirsty)	4 (20)	6 (30)	7 (35)	2 (10)	1 (5)	3 (18.8)	5 (31.3)	5 (31.30)	2 (12.5)	1 (6.3)	
	4. ฉันรู้สึกปากแห้งเมื่อฉันมีอาการกระหายน้ำ (My mouth feels dry when I am thirsty)	3 (15)	3 (15)	5 (25)	8 (40)	1 (5)	1 (6.3)	5 (31.3)	5 (31.3)	4 (25)	1 (6.3)	
	5. น้ำลายของฉันเหนียวมากเมื่อกระหายน้ำ (My saliva is very thick when I am thirsty)	3 (15)	5 (25)	6 (30)	5 (25)	1 (5)	2 (12.5)	5 (31.3)	4 (25)	5 (31.3)	0	
	6. เมื่อฉันดื่มน้ำน้อยลง อาการกระหายน้ำของฉันจะแย่ลง (When I drink less water, my thirst gets worse)	4 (20)	5 (25)	5 (25)	3 (15)	3 (15)	1 (6.3)	3 (18.8)	7 (43.8)	4 (25.0)	1 (6.3)	
	7. ฉันกระหายน้ำเป็นอย่างมาก จนฉันไม่สามารถควบคุมการดื่มน้ำได้ (I am so thirsty I could drink water uncontrollably)	5 (25)	7 (35)	4 (20)	3 (15)	1 (5)	3 (18.8)	6 (37.5)	4 (25)	3 (18.8)	0	
	8. อาการกระหายน้ำของฉันยากที่จะผ่านไปได้ (My thirst feels difficult to overcome)	6 (30)	7 (35)	7 (35)	0	0	4 (25)	6 (37.5)	5 (31.3)	1 (6.3)	0	
		Mean ± SD: 20.85 ± 7.17					Mean±SD: 21.56 ± 5.75					
		Cronbach’s α = 0.91					Cronbach’s α = 0.84					
SNAQ	1. ความอยากอาหารของฉัน (My appetite is)	1 (5)	0	9 (45)	4 (20)	6 (30)	0	0	6 (37.5)	7 (43.8)	3 (18.8)	First and second response Single measure 0.83 [95%CI 0.59–0.94] Average measure 0.91 [95% CI 0.74–0.97]
	2. เมื่อฉันทานอาหาร. . . (When I eat . . .)	1 (5)	0	2 (10)	16 (80)	1 (5)	0	0	3 (18.8)	12 (75)	1 (6.3)	
	3. รสชาติอาหาร. . . (Food tastes)	0	0	8 (40)	11 (55)	1 (5)	0	0	5 (31.3)	10 (62.5)	1 (6.3)	
	4. ฉันรับประทาน (Normally I eat)	0	1 (5)	8 (40)	11 (55)	0	0	0	5 (31.3)	10 (62.5)	1 (6.3)	
		Mean ± SD: 14.65 ± 2.30					Mean ± SD: 15.29 ± 1.46					
		Cronbach’s α = 0.71					Cronbach’s α = 0.46					

CI: confidence interval; ICC: intraclass correlation coefficients; SNAQ: Simplified Nutritional Appetite Questionnaire; SD: standard deviation; TDS-HF: Thirst Distress Scale for patients with heart failure.

The average measures of ICCs of TDS-HF and SNAQ were 0.910 [95% CI 0.74–0.97] and 0.910 [95% CI 0.74–0.97], respectively. The ranges of corrected item-total correlations of the TDS-HF and the SNAQ were reported as 0.55–0.79 and 0.37–0.78, respectively. These indicated that all items were correlated. In addition, if either item 3 or item 4 of the TDS-HF were deleted, which were perceived by some participants had having overlap, Cronbach’s α was calculated to show values of 0.90 and 0.89 respectively.

### Interview responses

The interview was conducted by the researcher (JT) and the duration of each interview ranged from 10 to 30 minutes. All participants reported that the instructions for both the TDS-HF and the SNAQ were comprehensible; in that, they understood what they were required to do and how to rate their thirst and appetite. About 90.0% of participants (*n* = 18) reported no issues with the translated TDS-HF, with only two participants reporting that they could not distinguish between Item 3 and Item 4.

Question 3 and 4, both are quite similar . . . dry mouth like a sandpaper and feeling dry mouth when having thirst . . . are the questions the same? (P2)

I feel like question 3 and 4 are quite similar (P3)

Furthermore, all participants reported understanding item 6 of the TDS-HF while 25.0% of those suggested that it does not reflect the way that Thai people would express their thirst. The original item says, ‘When I drink less water, my thirst gets worse’, but interviewees felt that Thai people would rather say ‘when I drink less water, I feel thirstier’.

I understand the sentence, but I feel like it is not natural as it is in a written form ummm do you understand . . . that it is not how we speak (P1)

The question is understandable, but if it would be more understandable, it would be better. I would suggest you change from ‘When I drink less water, my thirst gets worse’ to ‘I feel thirstier when I drink less water. (P20)

Additionally, 90.0% of participants reported that item 8 of the TDS-HF was comprehensible after the changes made after expert panel’s suggestions with the remaining participants suggesting that it was difficult to understand.

My thirst is difficult to overcome . . . hmmmm . . . difficult to overcome? The question is not designed to be easily understandable for lay people. It sounds like academic writing, but it is not how we speak that we can easily understand. This word . . . when I read, I feel strange. Difficult to overcome to me, this word uses in competition. (P1)

My thirst is difficult to overcome. What does this mean? . . . I need to drink water, so I feel better? (P3)

Participants reported understanding all items in the SNAQ without explanation, and there were no issues with wording or meaning in the items in this scale.

## Discussion

This study demonstrates preliminary reliability and validity of the Thai versions of the TDS-HF and the SNAQ. The thirst intensities were reported moderate for both test and retest. However, as the study was conducted during summer in Thailand, participants reported that they rated higher thirst intensity than usual. The results show that these instruments are reliable and valid with high Cronbach’s α at initial test and the confirmed face and content validities.

Cronbach’s α of the TDS-HF in the test and retest versions were 0.91 and 0.84, respectively. These findings are broadly in line with Cronbach’s α reported for translations of the TDS-HF in Swedish, German and Spanish (Eng et al., 2021; Waldréus et al., 2018b; Wefer et al., 2020). The Cronbach’s α values of the SNAQ in the test and retest were 0.71 and 0.46, respectively. The decreased Cronbach’s α in retest of SNAQ might be due to the return rate and the low number of test questions (Tavakol and Dennick, 2011). Additionally, insufficient inter-individual variability, such as subject fluctuation, could contribute to decreased Cronbach’s α in the SNAQ retest (Matheson, 2019). Inter-individual variability could have occurred due to the retest questionnaires subsequently being completed at home rather than in the clinic. For example, differences in distraction levels, temperature, and humidity between home settings and the clinic may have particularly affected perceptions of thirst. Future study designs should take these issues into consideration. Indeed, the SNAQ achieved the desired ICC levels. Similarly, the SNAQ translated from English to Japanese and German reported Cronbach’s α values of 0.545 and 0.64, and ICC values of 0.754 and 0.95, respectively (Nakatsu et al., 2015; Thai et al., 2023).

Qualitative findings regarding the TDS-HF and SNAQ, focusing on interpretation, comprehensibility, and linguistic and cultural relevance, were explored through an expert panel for content validity and semi-structured interviews for face validity. The assessment of content and face validity following the translation of these instruments into a new language has been extensively used for the TDS-HF and SNAQ (Eng et al., 2021; Waldréus et al., 2018b; Wefer et al., 2020; Thai et al., 2023). This approach is essential for patient-reported outcome measures, as it ensures that the translation is both clear and meaningful, while also accounting for cross-cultural differences (Wild et al., 2005).

Notably, interviews with participants revealed minimal issues with wording and confirmed good face validity. In addition, although some participants reported that item 3 and item 4 of the TDS-HF seem to be similar, the percentage who reported this issue was low. Equally, reliability testing with one or other of these questions deleted appeared to support the inclusion of both items.

### Strengths and limitations

The Thai versions of the TDS-HF and the SNAQ were translated following a robust forward–backward approach, their acceptability tested with a HF population and their reliability assessed. Addressing the lack of appropriate tools, these newly translated versions can eventually be used in clinical practice to assess thirst and appetite to facilitate HF management in Thailand. As nurses can play vital roles in clinical practice, having these two instruments available in the Thai language can assist nurses to promptly assess and manage thirst and appetite in the HF patients leading to an improvement in the quality of life and life satisfaction for individuals living with HF.

The SNAQ is a useful screening tool for cardiac cachexia in clinical practice (Evans et al., 2008; Thanapholsart et al., 2023). It will also support future empirical studies in a clinical area which is currently under-researched in Thailand due in part to an absence of suitable measures. As the tools to screen for cardiac cachexia are now available, it is crucial to promote screening for this disease through appropriate health policies. This is because once cardiac cachexia is diagnosed, the chances of fatality increase by 50% within 18 months (Anker et al., 1997).

In terms of limitations, this study adopted a convenience sampling approach, which may impact the generalisation of the results. In addition, the return rates of the retest TDS-HF and the SNAQ were lower than those completed in clinic, although the sample remained adequate for the study purposes. The study only included patients who were relatively stable. Thus, severely unwell patients, who may have had difficulties participating due to tiredness and breathing difficulties, were not represented, and may have interpreted questions differently. Finally, this was an initial study to confirm the accuracy of the tool translations and the reliability of items. In addition, concurrent validity was not conducted at this stage. It will be important in the future to conduct a broader range of psychometric tests with larger sample sizes; for example, the concurrent validity of these newly translated scales could be tested alongside the thirst visual analogue scale (Wefer et al., 2020; Waldréus et al., 2018b; Eng et al., 2021) and the Mini-Nutritional Assessment-Short Form (Sukkriang and Somrak, 2021; Nakatsu et al., 2015). Furthermore, there is a need for health policy to support and strengthen the implementation of the TDS-HF and the SNAQ, there is currently a lack of health policy to support this.

## Conclusion and recommendations

This study demonstrates adequate reliability and good content and face validity for the newly translated versions of the TDS-HF and the SNAQ in the Thai language. With further psychometric testing, these two instruments have the potential to benefit clinical practice and further research addressing the management of these frequent symptoms in people with HF. The availability of these two instruments not only benefit patients but also nurses and other health professionals in effectively assessing thirst and appetite ameliorating HF management. A well-defined health policy shall be put in place to ensure that the use of these instruments becomes a standard practice in healthcare settings.

**Key points for policy, practice and/or research**

- The Thai versions of the TDS-HF and the SNAQ are now available.
- The Thai versions of the TDS-HF and the SNAQ can be used in busy clinical settings as they take less than 5 minutes to complete.
- The Thai versions of the TDS-HF and the SNAQ can be used in chronic HF patients to assess thirst and appetite to facilitate HF management, including nursing management and care.
- The Thai versions of the TDS-HF and the SNAQ can be used in further research to assess thirst and appetite, including cachexia.
- The successful implementations of the Thai versions of the TDS-HF and the SNAQ requires a health policy that provides a framework for nurses and healthcare providers to incorporate the TDS-HF and SNAQ within their routine care for HF patients.

## Supplemental Material

sj-pdf-1-jrn-10.1177_17449871241292563 – Supplemental material for The assessment of reliability and validity of the Thai Versions of the Thirst Distress Scale for patients with Heart Failure and the Simplified Nutritional Appetite Questionnaire in heart failure patients Supplemental material, sj-pdf-1-jrn-10.1177_17449871241292563 for The assessment of reliability and validity of the Thai Versions of the Thirst Distress Scale for patients with Heart Failure and the Simplified Nutritional Appetite Questionnaire in heart failure patients by Jenjiratchaya Thanapholsart, Ehsan Khan, Satit Janwanishstaporn, Porntipa Thongma, Saowanee Naowapanich, Pornpoj Pramyothin, Srisakul Chirakarnjanakorn, Porntera Sethalao, Thitipong Tankumpuan, Nana Waldréus and Geraldine A. Lee in Journal of Research in Nursing

sj-pdf-2-jrn-10.1177_17449871241292563 – Supplemental material for The assessment of reliability and validity of the Thai Versions of the Thirst Distress Scale for patients with Heart Failure and the Simplified Nutritional Appetite Questionnaire in heart failure patients Supplemental material, sj-pdf-2-jrn-10.1177_17449871241292563 for The assessment of reliability and validity of the Thai Versions of the Thirst Distress Scale for patients with Heart Failure and the Simplified Nutritional Appetite Questionnaire in heart failure patients by Jenjiratchaya Thanapholsart, Ehsan Khan, Satit Janwanishstaporn, Porntipa Thongma, Saowanee Naowapanich, Pornpoj Pramyothin, Srisakul Chirakarnjanakorn, Porntera Sethalao, Thitipong Tankumpuan, Nana Waldréus and Geraldine A. Lee in Journal of Research in Nursing

sj-pdf-3-jrn-10.1177_17449871241292563 – Supplemental material for The assessment of reliability and validity of the Thai Versions of the Thirst Distress Scale for patients with Heart Failure and the Simplified Nutritional Appetite Questionnaire in heart failure patients Supplemental material, sj-pdf-3-jrn-10.1177_17449871241292563 for The assessment of reliability and validity of the Thai Versions of the Thirst Distress Scale for patients with Heart Failure and the Simplified Nutritional Appetite Questionnaire in heart failure patients by Jenjiratchaya Thanapholsart, Ehsan Khan, Satit Janwanishstaporn, Porntipa Thongma, Saowanee Naowapanich, Pornpoj Pramyothin, Srisakul Chirakarnjanakorn, Porntera Sethalao, Thitipong Tankumpuan, Nana Waldréus and Geraldine A. Lee in Journal of Research in Nursing

sj-pdf-4-jrn-10.1177_17449871241292563 – Supplemental material for The assessment of reliability and validity of the Thai Versions of the Thirst Distress Scale for patients with Heart Failure and the Simplified Nutritional Appetite Questionnaire in heart failure patients Supplemental material, sj-pdf-4-jrn-10.1177_17449871241292563 for The assessment of reliability and validity of the Thai Versions of the Thirst Distress Scale for patients with Heart Failure and the Simplified Nutritional Appetite Questionnaire in heart failure patients by Jenjiratchaya Thanapholsart, Ehsan Khan, Satit Janwanishstaporn, Porntipa Thongma, Saowanee Naowapanich, Pornpoj Pramyothin, Srisakul Chirakarnjanakorn, Porntera Sethalao, Thitipong Tankumpuan, Nana Waldréus and Geraldine A. Lee in Journal of Research in Nursing
