# Supplementary material for: The assessment of reliability and validity of the Thai Versions of the Thirst Distress Scale for patients with Heart Failure and the Simplified Nutritional Appetite Questionnaire in heart failure patients
Source: J Res Nurs. 2024 Dec 14:17449871241292563. Online ahead of print. doi: 10.1177/17449871241292563 (PMC11645761; doi:10.1177/17449871241292563)
Supplement: sj-pdf-1-jrn-10.1177_17449871241292563 – Supplemental material for The assessment of reliability and validity of the Thai Versions of the Thirst Distress Scale for patients with Heart Failure and the Simplified Nutritional Appetite Questionnaire in heart failure patients [file sj-pdf-1-jrn-10.1177_17449871241292563.pdf]

<table>
<tr><td>คำแปล <u>ฉบับ 1 (Matt)</u></td><td>คำแปล <u>ฉบับ 1 (Dean)</u></td><td>คำแปล <u>ฉบับ 2 (Matt)</u></td><td>คำแปล <u>ฉบับ 2 (Dean)</u></td></tr>
<tr><td colspan="4"><u>Original English version</u>
<u>Thirst Distress Scale for patients with heart failure (TDS-HF)</u>
Below you will find statements about your experience of thirst during the last 2-3 days. Read each statement carefully. Choose one of five possible answers for each statement that best describes your experience of thirst between strongly disagree (number 1) and strongly agree (number 5). Mark your chosen number with a circle.</td></tr>
<tr><td colspan="2">Forward translation <u>ฉบับ 1 (ชื่อแบบสอบถาม):</u>
แบบประเมินอาการกระหายน้ำและผลกระทบจากการกระหายน้ำในผู้ป่วยโรคหัวใจล้มเหลว</td><td colspan="2">Forward translation <u>ฉบับ 2 (ชื่อแบบสอบถาม):</u>
คะแนนความวิตกของการกระหายน้ำ สำหรับผู้ป่วยภาวะหัวใจล้มเหลว</td></tr>
<tr><td>Evaluation form – thirst and effects of thirst on congestive heart failure</td><td>Thirst assessment form and effects from thirst in patients with heart failure</td><td>Scoring concern about thirst for patients with congestive heart failure</td><td>Thirst scores for heart failure patients</td></tr>
<tr><td colspan="2">Forward translation <u>ฉบับ 1 (คำอธิบายการกรอกแบบสอบถาม):</u>
คำชี้แจง:
ข้อความด้านล่างนี้เป็นคำถามเกี่ยวกับประสบการณ์กระหายน้ำในช่วง 2-3 วันที่ผ่านมา
โปรดอ่านคำถามอย่างละเอียด และเลือก 1 คำตอบใน 5 ตัวเลือก ที่อธิบายประสบการณ์การกระหายน้ำของคุณได้ดีที่สุด ระหว่าง ไม่เห็นด้วยอย่างยิ่ง(1) และ เห็นด้วยอย่างยิ่ง(5)</td><td colspan="2">Forward translation <u>ฉบับ 2 (คำอธิบายการกรอกแบบสอบถาม):</u>
ท่านจะพบข้อความด้านล่างที่กล่าวถึงประสบการณ์ความกระหายน้ำของท่านในช่วง 2-3 วันที่ผ่านมา โปรดอ่านแต่ละประโยคอย่างละเอียด และเลือกคำตอบหนึ่งข้อที่อธิบายประสบการณ์ความกระหายน้ำของท่านได้ดีที่สุดจากทั้งหมด 5 ข้อ เรียงลำดับตั้งแต่ ไม่เห็นด้วยอย่างยิ่ง (หมายเลข 1) ถึง เห็นด้วยอย่างยิ่ง (หมายเลข 5)
โปรดวงกลมเลขที่ท่านเลือก</td></tr>
</table>

Text in RED and PURPLE are Backward Translation

โดยให้ท่านทำเครื่องหมายวงกลมหมายเลขที่ท่านเลือก			
Directions: the items below are questions about your experience with thirst in the last 2-3 days. Please read the questions carefully and choose 1 of the 5 options that best explain your experience with thirst, between ‘completely disagree (1) and completely agree (5) by circling the number you choose.	Explanation: The text below are questions about thirst experience over the past 2 - 3 day period. Please read the questions carefully and choose 1 answer amongst the 5 choices that best explains your experience of thirst between strongly disagree (1) and strongly agree (5) by marking the number you select.	You will see statements below mentioning your experience of thirst in the past 2-3 days. Please read each statement carefully and choose the answer that best explains your experience with thirst from 5 rankings: completely disagree (1) to completely agree (5) by circling the number you choose.	You will meet a statement below that discusses you thirst over the period of the past 2-3 days. Please read the sentence information thoroughly and choose one of five answers that best describes your thirst. Sort from strongly disagree (number 1) to strongly agree (number 5). Please circle the number you choose.

คำแปล ฉบับ 1 (Matt)	คำแปล ฉบับ 1 (Dean)	คำแปล ฉบับ 2 (Matt)	คำแปล ฉบับ 2 (Dean)

Text in RED and PURPLE are Backward Translation

<table>
<tr><td colspan="4"><u>Original English version</u>
1. My thirst bothers me a lot 2. I am very uncomfortable when I am thirsty 3. My mouth feels like sandpaper when I am thirsty 4. My mouth feels dry when I am thirsty 5. My saliva is very thick when I am thirsty 6. When I drink less water, my thirst gets worse 7. I am so thirsty I could drink water uncontrollably
8. My thirst feels difficult to overcome</td></tr>
<tr><td colspan="2">Forward translation <u>ฉบับ 1 (ข้อความในแบบสอบถาม):</u> 1. อาการกระหายน้ำรบกวนฉันเป็นอย่างมาก
2. ฉันรู้สึกไม่สุขสบายอย่างมากเมื่อฉันกระหายน้ำ
3.
ปากของฉันรู้สึก~~แห้ง~~สาก(เหมือนกระดาษทราย)เมื่อฉันมีอาการกระหายน้ำ
4. ฉันรู้สึกปากแห้งเมื่อฉันมีอาการกระหายน้ำ
5. น้ำลายของฉันเหนียวข้นมากเมื่อฉันมีอาการกระหายน้ำ
6. เมื่อฉันดื่มน้ำน้อยลง อาการกระหายน้ำของฉันจะแย่ลง
7. ฉันกระหายน้ำเป็นอย่างมาก จนฉันไม่สามารถควบคุมการดื่มน้ำได้
8. ฉันรู้สึกว่าอาการกระหายน้ำของฉันยากที่จะเอาชนะ</td><td colspan="2">Forward translation <u>ฉบับ 2 (ข้อความในแบบสอบถาม):</u> 1. ความกระหายน้ำของฉันก่อกวนใจอย่างมาก
2. ฉันรู้สึกอึดอัดไม่สบายใจเมื่อกระหายน้ำ
3. ปากของฉันรู้สึกเหมือนกระดาษทรายเมื่อกระหายน้ำ
4. ปากของฉันแห้งเมื่อกระหายน้ำ
5.น้ำลายของฉันเหนียวมากเมื่อกระหายน้ำ
6.เมื่อฉันดื่มน้ำน้อยกว่าปกติ ยิ่งทำให้รู้สึกกระหาย
7.ฉันรู้สึกกระหายน้ำมากถึงขั้นที่ฉันดื่มน้ำแบบควบคุมไม่ได้
8.อาการกระหายน้ำของฉันแก้ได้ยาก</td></tr>
<tr><td>1. Thirst bothers me a lot.
2. I don’t feel very comfortable when I’m thirsty.
3. My mouth feels dry like</td><td>1. My thirst symptoms bother me a lot
2. I feel not happy when I’m thirsty</td><td>1. My thirst bothers me a lot.
2. I feel uncomfortable and anxious when I’m thirsty.
3. My mouth feels dry like</td><td>1. My thirst is very disruptive
2. I feel uncomfortable when thirsty
3. My mouth feels like</td></tr>
</table>

Text in RED and PURPLE are Backward Translation

sandpaper when I am thirsty.<br>4. My mouth feels dry when I am thirsty.<br>5. My saliva is very sticky and thick when I am thirsty.<br>6. When I drink less water, my thirst gets worse.<br>7. I get very thirsty to the point where I can’t control how much water I drink.<br>8. I feel that my thirst is so bad, it’s too hard to overcome.	3. My mouth feels dry (like sandpaper) when I’m thirsty<br>4. I have a dry mouth when I have symptoms of thirst<br>5. My saliva is very sticky when I’m thirsty<br>6. When I drink less water, my thirst gets worse<br>7. I feel so thirsty, I am unable to control my drinking<br>8. I feel that my thirst is too hard to overcome	sandpaper when I am thirsty.<br>4. My mouth feels dry when I feel thirsty.<br>5. My saliva is very sticky when I feel thirsty.<br>6. When I drink less water than usual, it makes me feel more thirsty.<br>7. I get very thirsty to the point where I can’t control how much water I drink.<br>8. My thirst problem cannot be solved.	sandpaper when thirsty<br>4. My mouth is dry when thirsty<br>5. My saliva is very sticky when thirsty<br>6. When I drink less water than normally, it makes me feel thirsty<br>7. I feel very thirsty that I drink out of control<br>8. My thirst symptoms are hard to solve

Text in RED and PURPLE are Backward Translation

แบบประเมินอาการกระหายน้ำและผลกระทบจากการกระหายน้ำในผู้ป่วยโรคหัวใจล้มเหลว

คำชี้แจง:
“ท่านจะพบข้อความด้านล่างที่กล่าวถึงประสบการณ์ความกระหายน้ำของท่านในช่วง 2-3 วันที่ผ่านมา โปรดอ่านแต่ละประโยคอย่างละเอียด และเลือก 1 คำตอบใน 5 ตัวเลือก ที่อธิบายประสบการณ์การกระหายน้ำของคุณได้ดีที่สุด ระหว่าง ไม่เห็นด้วยอย่างยิ่ง(1) และ เห็นด้วยอย่างยิ่ง(5) โดยให้ท่านทำเครื่องหมายวงกลมหมายเลขที่ท่านเลือก”

	**ไม่เห็นด้วยอย่างยิ่ง (1)**	**ไม่เห็นด้วย (2)**
1. อาการกระหายนำของฉันก่อกวนใจอย่างมาก		
2. ฉันรู้สึกไม่สุขสบายอย่างมากเมื่อฉันกระหายน้ำ		
3. ปากของฉันรู้สึกแห้งสาก (เหมือนกระดาษทราย)เมื่อฉันมีอาการกระหายน้ำ		
4.ฉันรู้สึกปากแห้งเมื่อฉันมีอาการกระหายน้ำ		
5.นำลายของฉันเหนียวมากเมือกระหายนำ		
6. เมื่อฉันดื่มน้ำน้อยลง อาการกระหายน้ำของฉันจะแย่ลง		
7. ฉันกระหายน้ำเป็นอย่างมาก จนฉันไม่สามารถควบคุมการดื่มน้ำได้		
8.อาการกระหายน้ำของฉันยากที่จะผ่านไปได้		

Text in RED and PURPLE are Backward Translation

Text in RED and PURPLE are Backward Translation
