# Supplementary material for: The assessment of reliability and validity of the Thai Versions of the Thirst Distress Scale for patients with Heart Failure and the Simplified Nutritional Appetite Questionnaire in heart failure patients
Source: J Res Nurs. 2024 Dec 14:17449871241292563. Online ahead of print. doi: 10.1177/17449871241292563 (PMC11645761; doi:10.1177/17449871241292563)
Supplement: sj-pdf-2-jrn-10.1177_17449871241292563 – Supplemental material for The assessment of reliability and validity of the Thai Versions of the Thirst Distress Scale for patients with Heart Failure and the Simplified Nutritional Appetite Questionnaire in heart failure patients [file sj-pdf-2-jrn-10.1177_17449871241292563.pdf]

<table>
<tr><td>คำแปล <u>ฉบับ 1 (Matt)</u></td><td>คำแปล <u>ฉบับ 1 (Dean)</u></td><td>คำแปล <u>ฉบับ 2 (Matt)</u></td><td>คำแปล <u>ฉบับ 2 (Dean)</u></td></tr>
<tr><td colspan="4">Simplified Nutritional Appetite Questionnaire
Instruction: The following questions refer to your appetite. Please read and complete the following questions. Please mark the answer that best applies to you.</td></tr>
<tr><td colspan="2">Forward translation <u>ฉบับ 1</u>:
แบบประเมินความอยากอาหาร</td><td colspan="2">Forward translation <u>ฉบับ 2</u>:
แบบสอบถามความอยากอาหาร ฉบับง่าย</td></tr>
<tr><td>Evaluation form – appetite</td><td>Assessment form for appetite</td><td>Survey on appetite - simplified</td><td>Appetite Assessment Form Simple</td></tr>
<tr><td colspan="2">Forward translation <u>ฉบับ 1</u>:
คำชี้แจง: คำถามต่อไปนี้เกี่ยวข้องกับความอยากอาหารของท่าน กรุณาอ่านและตอบคำถามโดยวงกลมเลือกคำตอบที่ตรงกับท่านมากที่สุด</td><td colspan="2">Forward translation <u>ฉบับ 2</u>:
คำชี้แจง คำถามต่อไปนี้เกี่ยวข้องกับความอยากอาหารของท่าน
โปรดอ่านและตอบคำถามดังกล่าว
โปรดวงกลมคำตอบที่อธิบายกรณีของท่านได้ใกล้เคียงที่สุด</td></tr>
<tr><td>Directions: The questions below concern your appetite. Please read and answer them by circling the answer most applicable to you.</td><td>Explanation. The questions here are about your appetite. Please read and answer the questions by circling the answer that best suits you.</td><td>Directions: The questions below are about your appetite. Please read and answer them by circling the answer that applies most closely to you.</td><td>Explanation. The questions here are about your appetite. Please read and answer. Please circle the answer that describes your case as closely as possible</td></tr>
</table>

Text in RED and PURPLE are backward translation.

Original English version			
1. My appetite is<br>a. very poor b. poor<br>c. average<br>d. good<br>e. very good	2. When I eat<br>a. I feel full after eating only a few mouthfuls b. I feel full after eating about a third of a meal<br>c. I feel full after eating over half a meal d. I feel full after eating most of the meal e. I hardly ever feel full	3. Food tastes<br>a. very poor<br>b. poor<br>c. average<br>d. good e. very good	4. Normally I eat<br>a. less than one meal a day<br>b. one meal a day<br>c. two meals a day<br>d. three meals a day<br>e. more than three meals a day
Forward translation <u>ฉบับ1</u>:<br>1. ความอยากอาหารของฉัน	2. เมื่อฉันทานอาหาร…<br>ก. ฉันรู้สึกอิ่มหลังทานอาหารไม่กี่คำ<br>ข. ฉันรู้สึกอิ่มหลังจากทานอาหารได้ 1 ใน 3 ของมื้ออาหาร<br>ค. ฉันรู้สึกอิ่มหลังจากทานอาหารได้เกินครึ่งนึงของมื้ออาหาร<br>ง. ฉันรู้สึกอิ่มหลังจากทานอาหารได้เกือบทั้งหมด จ. ฉันแทบจะไม่รู้สึกอิ่ม	3. รสชาติอาหาร …<br>ก. แย่มาก ข. แย่<br>ค. ปานกลาง<br>ง. ดี จ.	4. โดยปรกติฉันทานอาหาร …<br>ก. น้อยกว่า1มื้อต่อวัน ข. 1 มื้อต่อวัน<br>ค. 2 มื้อต่อวัน ง. 3 มื้อต่อวัน<br>จ. มากกว่า 3 มื้อต่อวัน

Text in RED and PURPLE are backward translation.

ก. แย่มาก ข. แย่<br><br>ค.ปานกลาง<br>ง.ดี จ.ดีมาก		ดีมาก	
**คำแปล ฉบับ 1**<br>**(Matt)**<br>My appetite is…<br>a. very poor<br>b. poor<br>c. neither good nor bad<br>d. good<br>e. very good	When I eat…<br>a. I feel full after just a few bites. b. I feel full after 1/3rd of a meal<br>c. I feel full after ½ of a meal d. I feel full after eating almost everything on my plate.<br>e. I feel barely full from eating.	The taste of food is…<br>a. very poor<br>b. poor<br>c. neither good nor bad<br>d. good<br>e. very good	I usually eat<br>a. less than one meal per day<br>b. one meal per day<br>c. two meals per day<br>d. three meals per day<br>e. more than 3 meals per day
**คำแปล ฉบับ 1**<br>**(Dean)**<br>My appetite is..<br>a. Very bad b. Bad	When I eat..<br>a. I feel full after a few bits of food b. I feel full after eating a 1/3 of the meal<br>c. I feel full after eating more than half the meal d. I feel	Food tastes…<br>a. Very bad b. Bad<br>c. Moderate	Typically, I eat…<br>a. Less than 1 meal a day<br>b. 1 meal per day<br>c. 2 meals per day

Text in RED and PURPLE are backward translation.

c. Moderate<br>d. good<br>e. very good	full after eating most of the meal<br>e. I've never felt full	d. good<br>e. very good	d. 3 meals per day<br>e. More than 3 meals per day

Original English version			
1. My appetite is<br>a. very poor b. poor<br>c. average d. good<br>e. very good	2. When I eat<br>a. I feel full after eating only a few mouthfuls b. I feel full after eating about a third of a meal<br>c. I feel full after eating over half a meal d. I feel full after eating most of the meal e. I hardly ever feel full	3. Food tastes<br>a. very poor<br>b. poor<br>c. average<br>d. good e. very good	4. Normally I eat<br>a. less than one meal a day<br>b. one meal a day<br>c. two meals a day<br>d. three meals a day<br>e. more than three meals a day
Forward translation ฉบับ2:<br>1. ความอยากอาหารของฉัน	2. เมื่อฉันรับประทานอาหาร…<br>ก. ฉันรู้สึกอิ่มหลังจากรับประทานเพียงไม่กี่คำ<br>ข. ฉันรู้สึกอิ่มหลังจากรับประทานไปหนึ่งในสามของมื้อ<br>ค. ฉันรู้สึกอิ่มหลังจากรับประทานไปเกินครึ่งหนึ่งของมื้อ<br>ง. ฉันรู้สึกอิ่มหลังจากรับประทานไปเกือบทั้งหมด	3. อาหารมีรสชาติ<br>ก. แย่มาก ข. แย่<br>ค. ปานกลาง	4. ฉันรับประทาน<br>ก. น้อยกว่าหนึ่งมื้อต่อวัน<br>ข. หนึ่งมื้อต่อวัน<br>ค. สองมื้อต่อวัน<br>ง. สามมื้อต่อวัน

Text in RED and PURPLE are backward translation.

ก. แย่มาก ข. แย่<br>ค.ปานกลาง<br>ง.ดี จ.ดีมาก	จ. ฉันไม่ค่อยรู้สึกอิ่ม	ง. ดี จ.<br>ดีมาก	จ. มากกว่าสามมื้อต่อวัน
คำแปล ฉบับ 2<br>(Matt)<br>Similar to 1st version above	When I eat…<br>a. I feel full after just a few bites. b. I feel full after 1/3rd of a meal<br>c. I feel full after ½ of a meal d. I feel full after eating almost all of my meal.<br>e. I don't really feel full.	Similar to 1stversion above	Similar to 1stversion above
คำแปล ฉบับ 2<br>(Dean)<br>Similar to 1st version above	When I eat..<br>a. I feel full after eating a little b. I feel full after eating a 1/3 of the meal<br>c. I feel full after eating a half of the meal d. I feel full after eating almost all of the meal<br>e. I don't feel full	Food flavour is …<br>a. Very bad<br>b. Bad<br>c. Moderate	Normally, I eat …<br>a. Less than one meal a day<br>b. One meal a day<br>c. Two meals a day<br>d. 3 meals a day<br>e. More than 3 meals a day

Text in RED and PURPLE are backward translation.

		d. good<br>e. very good	

Text in RED and PURPLE are backward translation.

แบบสอบถามความอยากอาหาร ฉบับง่าย

คำชี้แจง: คำถามต่อไปนี้เกี่ยวข้องกับความอยากอาหารของท่าน กรุณาอ่านและตอบคำถามโดยวงกลมเลือกคำตอบที่ตรงกับท่านมากที่สุด

1. ความอยากอาหารของฉัน

ก. แย่มาก
ข. แย่
ค.ปานกลาง
ง.ดี

จ.ดีมาก

2. เมื่อฉันทานอาหาร…

ก. ฉันรู้สึกอิ่มหลังทานอาหารไม่กี่คำ

ข. ฉันรู้สึกอิ่มหลังจากทานอาหารได้ 1 ใน 3 ของมื้ออาหาร

ค. ฉันรู้สึกอิ่มหลังจากทานอาหารได้เกินครึ่งนึงของมื้ออาหาร

ง. ฉันรู้สึกอิ่มหลังจากทานอาหารได้เกือบทั้งหมด จ. ฉันแทบจะไม่รู้สึกอิ่ม

3. รสชาติอาหาร ...

ก. แย่มาก ข. แย่
ค. ปานกลาง
ง. ดี จ. ดีมาก

4. ฉันรับประทาน

ก. น้อยกว่าหนึ่งมื้อต่อวัน
ข. หนึ่งมื้อต่อวัน
ค. สองมื้อต่อวัน
ง. สามมื้อต่อวัน
จ. มากกว่าสามมื้อต่อวัน

Text in RED and PURPLE are backward translation.
