# Supplementary material for: The assessment of reliability and validity of the Thai Versions of the Thirst Distress Scale for patients with Heart Failure and the Simplified Nutritional Appetite Questionnaire in heart failure patients
Source: J Res Nurs. 2024 Dec 14:17449871241292563. Online ahead of print. doi: 10.1177/17449871241292563 (PMC11645761; doi:10.1177/17449871241292563)
Supplement: sj-pdf-4-jrn-10.1177_17449871241292563 – Supplemental material for The assessment of reliability and validity of the Thai Versions of the Thirst Distress Scale for patients with Heart Failure and the Simplified Nutritional Appetite Questionnaire in heart failure patients [file sj-pdf-4-jrn-10.1177_17449871241292563.pdf]

แบบสอบถามความอยากอาหาร ฉบับง่าย

คำชี้แจง: คำถามต่อไปนี้เกี่ยวข้องกับความอยากอาหารของท่าน กรุณาอ่านและตอบคำถามโดยวงกลมเลือกคำตอบที่ตรงกับท่านมากที่สุด

1. ความอยากอาหารของฉัน

ก. แย่มาก ข. แย่ ค.ปานกลาง ง.ดี จ.ดีมาก

2. เมื่อฉันรับประทานอาหาร…

ก. ฉันรู้สึกอิ่มหลังรับประทานอาหารไม่กี่คำ

ข. ฉันรู้สึกอิ่มหลังจากรับประทานอาหารได้ 1 ใน 3 ของมื้ออาหาร

ค. ฉันรู้สึกอิ่มหลังจากรับประทานอาหารได้เกินครึ่งนึงของมื้ออาหาร

ง. ฉันรู้สึกอิ่มหลังจากรับประทานอาหารได้เกือบทั้งหมด

จ. ฉันแทบจะไม่รู้สึกอิ่ม

3. รสชาติของอาหาร ...

ก. แย่มาก ข. แย่ ค.ปานกลาง ง.ดี จ.ดีมาก

4. โดยปรกติฉันรับประทานอาหาร …

ก. น้อยกว่า1มื้อต่อวัน

ข. 1 มื้อต่อวัน

ค. 2 มื้อต่อวัน

ง. 3 มื้อต่อวัน

จ. มากกว่า 3 มื้อต่อวัน
